# 

**Published:** 1877-07

**Authors:** 


### Contents of July Number.

### ORIGINAL.

ART. I.—Feasibility of Extirpating the Thyroid Gland in some cases of Disease, with report of Case, by J. F. Miner, M. D....... 323
ART. II.—On Albumen in the treatment of Pulmonary Consumption, by E. L. Shurly, M. D., Detroit, Mich., Concluded ....... ... 327
ART. III.—Laceration of Duodenum, with Post Mortem, E. V. Stoddard, M. D........................................ 337

### MISCELLANEOUS.

The State Medical Society...... ........ .................... 338
Relation of Air to the House we Live in, by Dr. Max Von Pettenkofer....... ........................................... 343
Case of Membranous Croup—New Method of Treatment—Recovery, by A. Fulton, M. D.......... ........................... 345
A Lost Art in Surgery, by A. B. Crosby, M. D.................... 346
Disinfecting Oven in Mercy Hospital, by E. Andrews, M. D.......... 346
Health of Cities..... ... ......................................... 348
Safe and Rapid Cure for Aneurism.................................. 348
The Production of Albuminuria........... ....................... 348

### EDITORIAL.

Volume XVI.................................................. 349
American Medical Association........................................ 349
Women's Hospital.................... ....... ......... ... . 350
Dialysed Iron.................... ................ ....... 351
American Dermatological Association........ .. .. . ............ 351
The Popular Science Monthly.... ............................... 351

Books Reviewed:

Myelitis of the Anterior Horns........ ................ ... 352
Annual Report U. S. M. Hosp. Service for 1875............ 352
The Practitioner's Hand-Book of Treatment. ............ 352
Contributions to Reparative Surgery................. ...... 353
A Course of Practical Histology. .......... ............... 354
A Directory for the Dissection of the Human Body......... 354
The Microscope...... ...................... .......... 355

Books and Pamphlets Received................................... 355
INDEX TO VOLUME XVI.

